# Inland capture fisheries

**DOI:** 10.1098/rstb.2010.0168

**Published:** 2010-09-27

**Authors:** Robin L. Welcomme, Ian G. Cowx, David Coates, Christophe Béné, Simon Funge-Smith, Ashley Halls, Kai Lorenzen

**Affiliations:** 1 Division of Biology, Imperial College London, Silwood Park, Ascot SL5 7PY, UK; 2 Hull International Fisheries Institute, University of Hull, Hull HU6 7RX, UK; 3 Secretariat of the Convention on Biological Diversity, 413 Saint Jacques Suite 800, Montreal, CanadaH2Y 1N9; 4 World Fish Center, Penang, Malaysia; 5 FAO Regional Office for Asia and the Pacific, 39 Pra Athit Road, Bangkok 10900, Thailand; 6 Aquae Sulis Limited (ASL), Midway House, Turleigh, Bradford-on-Avon, Wiltshire BA15 2LR, UK

**Keywords:** drivers, inland fisheries, lakes; rivers, social and economic issues, catch trends

## Abstract

The reported annual yield from inland capture fisheries in 2008 was over 10 million tonnes, although real catches are probably considerably higher than this. Inland fisheries are extremely complex, and in many cases poorly understood. The numerous water bodies and small rivers are inhabited by a wide range of species and several types of fisher community with diversified livelihood strategies for whom inland fisheries are extremely important. Many drivers affect the fisheries, including internal fisheries management practices. There are also many drivers from outside the fishery that influence the state and functioning of the environment as well as the social and economic framework within which the fishery is pursued. The drivers affecting the various types of inland water, rivers, lakes, reservoirs and wetlands may differ, particularly with regard to ecosystem function. Many of these depend on land-use practices and demand for water which conflict with the sustainability of the fishery. Climate change is also exacerbating many of these factors. The future of inland fisheries varies between continents. In Asia and Africa the resources are very intensely exploited and there is probably little room for expansion; it is here that resources are most at risk. Inland fisheries are less heavily exploited in South and Central America, and in the North and South temperate zones inland fisheries are mostly oriented to recreation rather than food production.

## 1. Introduction

Inland capture fisheries group activities that extract fish and other living organisms from surface waters inland of the coastline. In 2008, inland capture fisheries produced an estimated 10 million tonnes of fish and crustaceans (FAO Fishstat 2010—see http://www.fao.org/fishery/statistics/software/fishstat/en). As a valuable source of protein-rich food and employment, inland fisheries deliver nutritional security and income to hundreds of millions of rural households. Nevertheless, there are serious misperceptions about the magnitude, benefits and sustainability of inland fisheries resources which limit the effectiveness of national and international policies for their management and undermine their future.

Inland fisheries are dynamic. As economies evolve the nature of inland fisheries changes (Arlinghaus *et al*. 2002). The importance of high-value inland recreational fisheries grows and reliance on fisheries for food declines as local economies develop.

Inland fisheries are distinct from marine fisheries in their nature and in the range of drivers that influence them. Although commercially intensive fisheries exist, inland fisheries are generally characterized by small-scale/household-based activities. Participation in fisheries is high and the bulk of the catch is consumed locally. By-catch is insignificant as practically all fish caught are used. This means that their benefits are widely spread. Inland fisheries are also very diverse, being based on a range of ecosystems whose fish communities respond very differently to internal (fisheries-based) and external (natural- and human ecosystem-based) drivers.

One conceptual driver of inland fisheries is the widespread vision of inevitable demise of inland fisheries in the face of escalating human impacts, which is reflected by studies from all continents (Friend *et al*. 2009). Catches are allegedly falling, species disappearing and many other symptoms of chronic overfishing are reported. There is an assumption that overfishing is to blame, which is influenced by perceptions derived from marine fisheries. This instils a sense of hopelessness, fuelling neglect and subordination to agricultural, industrial and domestic sectors, particularly with respect to competing resources. The contribution of wild-caught, inland fish to food security has been largely ignored, and priorities switched to other sectors. Aquaculture is promoted as the means to maintain production in the face of this perceived decline, a view prominent throughout the tropics and widely held by aid agencies. The result is a lack of resources assigned to inland fisheries, a lack of information and apparent failure to incorporate inland fisheries' interests adequately into administrative structures. In addition, governments and resource developers see inland fisheries as an impediment to their desires to expropriate the wealth of the rivers—the transfer of generalized wealth (nutritional security, livelihoods) from powerless people into focused income streams that benefit powerful people (Osborne 2010).

**Figure 1.**
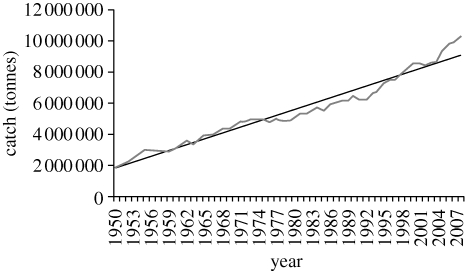
Global trends in inland fish catch 1950–2008—including fish, Crustacea and Mollusca, excluding reptiles and mammals (*y*=12311*x* + 2E+06; *r*^2^ = 0.960). From FAO Fishstat database.

Nevertheless, reported catches from inland fisheries are still rising at a linear rate of about 3 per cent per year globally (figure 1). There is widespread evidence that much of the catch from inland fisheries is unrecorded, partly because of the diffuse and small-scale nature of individual fisheries, the lack of easily definable landings, and because much of the catch goes directly to domestic consumption (e.g. Welcomme 1976, for rivers; Coates 2002, for Asia; Hortle *et al*. 2008, for rice fields; Braimah (in Béné 2007), for Volta Lake; Lymer *et al*. 2008, for Thailand). The Food and Agriculture Organization of the United Nations (FAO) itself posts caveats about the quality of the inland fisheries statistics in its SOFIA (The State of World Fisheries and Aquaculture) reviews (FAO 2002, 2004, 2007, 2009).

These contrasting views of increasing production and underestimation of the fisheries *versus* continuing reports of declining fish catches, loss of diversity and lack of potential in individual fisheries are difficult to reconcile because a lack of indicators hinders the formulation of management policy.

This review attempts to clarify some of the issues surrounding the various types of inland fishery to define their role in food security and in so doing defines the various drivers operating on inland fisheries, where drivers are defined as factors influencing yield, changes and sustainability in inland fish resources and fisheries.

## 2. Drivers regulating inland fisheries

Demand is the primary driver of almost any human activity including inland fisheries, aquaculture and marine fisheries. It also regulates water management, power supply, mining, forestry, agriculture or any of the other influences on inland waters. Demand operates through a series of more immediate drivers as described in table 1. This summarizes the principal drivers regulating inland fisheries, the mechanisms through which they operate, their effects and some solutions. Further details on some drivers are discussed in the sections listed.

**Table 1.** Summary of the principal drivers influencing yield, changes and sustainability in inland fish resources and fisheries.

driver	mechanism	effect	solution	section
*overall driver*				
demand	demand for food	increases pressure on fishery	on the whole demand is outside the control of the fisheries sector although many of the drivers arising from it are	
	demand for recreation	shifts objective of fishery from food to recreational fishing		
	demand for other services	described in detail below		
*direct drivers internal to the fishery: governance, regulation and management*				
governance, regulation and management	inadequate fisheries management infrastructure	inadequate legislation and enforcement mechanisms	improved administration and training of fisheries staff	8
excessive/illegal/unregulated fishing	open access to large numbers of fishers	overexploitation of individual species	control of access to fishery and of fisher numbers	5.1; 7.1
	proliferation of unregulated fishing and use of damaging gears	extreme fishing-down of fish assemblage	better stewardship of resources by stakeholders	
	overexploitation of some groups/sizes, underexploitation of others	falling catches of species of major economic value	regulation of fishing gears, protected areas and closed seasons	
		progressive decline in size of fish	encouragement of self-policing by fishing communities	
		declines in amount and value of catches	science-based policies and management	
		failure to obtain optimal yields from fishery		
fishery enhancement	introductions of alien species	increases in overall production	adherence to accepted guidelines for introductions and stocking	7; 8.3
	stocking	damage to existing fish stocks through competition and predation by introduced/stocked species		
		degradation of habitats		
		loss of biodiversity and disruption of genetic integrity		
*direct drivers external to the fishery:natural drivers*				
short-term climatic variation	periods of floods and drought	during droughts, declines in fishery production of rivers and river-controlled lakes and reservoirs	little can be done to control natural processes but some mitigation can occur by changing water withdrawal policies during times of drought	7
		during floods, increased abundance and productivity in floodplain rivers and associated water bodies		
		changes in species dominance of fish present		
*direct drivers external to the fishery: human-induced drivers*				
agricultural impacts on water resources	wetland drainage	reduces area available for fish especially in rivers/wetlands	land-use policies, opportunity for synergies with irrigated crops such as rice	7.2
	water abstraction	disrupts natural flow patterns	study and apply environmental flows	
	pollution	poisons fish and creates fishless lakes and zones in rivers	control point source effluent discharges	
	eutrophication	may increase productivity to a point but changes species composition	control fertilizer use and diffuse pollution sources integrated water resources management	
		
damming for hydropower generation, flood control and water supply	damming	disrupts/bars migration pathways of fish	install fish passage facilities where possible	7.2
	creation of impoundment upstream	loss of biodiversity and long distance migrating species	provision of downstream fish guidance systems	
	changes to flood regime	disrupts natural flow patterns	study and apply environmental flows	
		loss of spawning and nursery habitat upstream		
		change in ecosystem functioning	integrated water resources management	
	injury and loss of downstream migrants in turbines	severe losses of fishes in turbines		
forestry	changes to flood regime	disrupts natural flow patterns	deforestation and reforestation regulations	7.2
	increased siltation	alters aquatic ecosystem structure and functioning	integrated land-water resources management	
industrial and domestic water uses	water abstraction	disrupts natural flow patterns	study and apply environmental flows	7.2
	pollution	poisons fish and creates fishless lakes and zones in rivers	control point source effluent discharges	
	eutrophication	may increase productivity to a point but changes species composition	control sewage discharges and diffuse pollution from road run-offs	
		loss of biodiversity	integrated water resources management	
mining	pollution	poisons fish and creates fishless lakes and zones in rivers	control point source pollution and siltation through settling ponds	7b
	siltation	changes form of aquatic environment	integrated water resources management	
modification of river channels for flood control and navigation	changes to river channel form	changes form and function of aquatic environment	river/lake rehabilitation	7b
	dredging main channel of rivers	altered flow regimes	integrated water resources management	
	elimination of riparian and instream vegetation	disconnection of floodplain from river system		
	lock systems create barriers to fish migration	loss of biodiversity		
long-term climate change/global warming	failure of existing flood patterns	shifts in overall abundance of fish	global climate change control measures	10
	desiccation of some lakes	changes in species composition	formulation of long-term water use strategies that take possible changes into account	
	changes in thermal regimes			
*indirect drivers:political, social and economic drivers*				
population growth	increasing demands for food and resources (particularly water)	increases related direct drivers on inland fishery loss through focus on land-based food production	improved natural resources management planning	
unemployment	forces populations onto inland fisheries as resource of last resort	anarchic fishing and overexploitation	poverty reduction and employment strategies	
		increasing number of fishers exploiting finite inland fisheries		
lack of property rights for fishers	fishers' traditional use of the resource is not recognized by others wishing to use the water resource	water resource is used by other agents, reducing fish habitat and availability, displacement of fishers	legal recognition of traditional property rights	
shifts in consumer preferences	increasing consumption of water-intensive foods (particularly meat)	increases pressures on water resources	improved natural resources management planning	
	increasing preferences for fishery products	increases motivations for increased fisheries production	certification schemes for foods—including labelling	
policy objectives	low perceived priority of inland fisheries in provision of goods and services	influences priority awarded to inland fisheries in overall planning	better science-based advocacy for inland fisheries sector	
	choices made at government levels with regard to place of the aquatic environment and fisheries in overall land- and water-use policy	influences allocation of funds for management and research	improved adherence to principles of international agreements on environment	
			clear inclusion of inland fisheries in general land and water use planning	
failure of planning in international river/lake basins	uncoordinated research, monitoring and management of water resources	unsustainable water use	establishment of new or reinforcement of existing mechanisms for research and management of international inland waters	8.3
		failure of cross-boundary and migratory stocks	strengthen trans-boundary cooperation (e.g. adoption of existing trans-boundary institutional mechanisms)	
natural and man-made disasters/famine	lack of resilience in rural poor communities	anarchic fishing and overexploitation	better civil order disaster relief	
	forces populations onto inland fisheries as resource of last resort	inland fisheries underpin localized food security under post-disaster conditions	improved disaster mitigation planning	
			better ecosystem management for disaster reduction	
land distribution	creation of a landless population dependent on inland fisheries	increasing fishing pressure by dependent groups	better land distribution policies	
recreational fishing	shift from food fishing to recreational fishing	reduces proportion of resource available as food	water-use policies recognize at an early stage the importance of public pressures for increasing water quality and availability	7.1; 8.4
		recreational interests begin to influence environmental policies in particular regarding water use		
		agriculture no longer has a free rein regarding water use		
water-related human health issues	deteriorating environmental quality influences human health (e.g. water quality issues and proliferation of water-borne diseases)	increased demands for improved water quality and environmental health	improved integrated water resources management	
			ecosystem rehabilitation	

## 3. State of knowledge of the resource

### (a) State of statistics

Most countries report their inland fish catch statistics to FAO, where they are accessible through Fishstat (http://www.fao.org/fishery/statistics/software/fishstat/en).

Several weaknesses are apparent in the existing statistics including:

— inadequate data collections systems;
— selective data collection;
— double counting of landings;
— confusion with aquaculture; and
— political pressure.

Most countries do not specify their sampling and reporting procedures so it is difficult to compare results between countries. As a result many of the nominal catch statistics must be considered as unreliable and should not be used unless they are reconciled with other sources of information (Coates 2002). Where the errors and biases are considered constant, the statistics may be used to indicate trends (e.g. Lymer & Funge-Smith 2009).

### (b) Estimates of trends in inland fish catch

FAO nominal fish catch statistics reported a total catch of 10 220 499 tonnes in 2008 for the inland waters of the world. Catches have risen steadily at about 3.05 per cent per year since the beginning of FAO statistical records in 1950 (figure 1).

**Figure 2.**
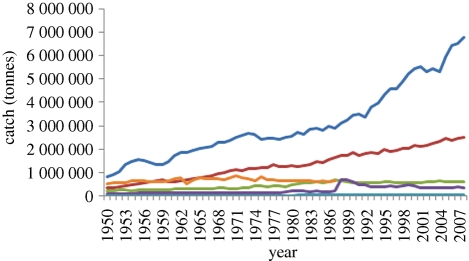
Trends in catch by continent 1950–2008 (dark blue, Asia; brown, Africa; green, Americas; violet, Europe; light blue, Oceania; yellow, ex USSR territories). From FAO Fishstat database. N.B. The FAO dataset is discontinuous for the old USSR countries which were reported as a group (other) until 1987. After that date they were split into individual reports. Here the catches from the old USSR including Russia were combined with those of Europe for a continuous dataset—inland water catches from the former Asian USSR republics are now generally negligible.

Trends in catch by continent suggest the main increases are associated with Asia and Africa, and to a lesser extent the Americas (figure 2). Table 2 shows percentage contribution by continent and the growth rate in catch over the last 10 years by continent. The declines in catch noted in Europe and North America can be attributed to the progressively greater use of inland fish resources for recreational fisheries.

**Table 2.** Inland water catch^a^ and relative contribution (percentage) for each continent to the global inland water catch in 2008 and the percentage growth rate in inland fisheries catches over the last 10 years. From FAO FishStat (2010).

continent	2008 (tonnes)	relative contribution (%)	mean % per year movement over last 10 years (1998–2008)
global total	10 220 451	100	2.73
Asia	6 786 534	66.40	3.48
Africa	2 502 570	24.49	2.29
America South	378 484	3.70	1.09
Europe	357 057	3.49	−1.10
America North and central	178 068	1.74	−1.41
Oceania	17 786	0.17	−1.24

^a^including all catch.

### (c) State of exploitation

Most inland fisheries are multi-species, multi-gear in nature, so standard assessment models and concepts of overfishing are inappropriate and can be applied only in a few lakes where a limited number of species are exploited by a homogeneous fishery. Instead, the fishing-down process that operates in many inland waters suggests that the main indicator of heavy fishing is a reduction of mean size (and age) of the fish landed. In many areas of the tropics, the mean size and age of the catch have reduced progressively over the years, until in some cases the major part of the catch consists of fish in the first year of life (see, for example, Lae 1995; Halls *et al*. 1999). In addition, fisher numbers have increased throughout Asia and Africa (see FAO database on fishermen numbers—http://www.fao.org/fishery/statistics/programme/3,1,1/en). These factors indicate that most inland fisheries in these continents are heavily fished to a degree that substantially alters the species composition, abundance and ecology of the fish communities, and that there is probably little room for any substantial increases in catch. Fishing pressures in South America do not appear to have reached these levels, as catches still include large species, and there is probably some room for increase. In other areas, catches appear to be maintained by stocking programmes. In the temperate zone, inland fisheries resources seem to be increasingly oriented towards recreation and conservation (Arlinghaus *et al*. 2002; Cowx *et al*. 2010), although there is growing evidence that recreational fisheries are having significant impacts of stocks both from fishing pressure and stock dynamics (Cooke & Cowx 2004, 2006).

### (d) Importance of inland fisheries

Fish from all sources form the major single source of animal protein worldwide, accounting for over one-third (36.58%) of global production in 2007 (table 3). Based on current statistical information, inland fisheries account for 2.36 per cent of animal protein sources. This figure is very likely to be underestimated as compared with their 6.8 per cent contribution to the world total fish production, as about 90 per cent of fish from inland capture is for human consumption as opposed to marine fisheries where a substantial amount goes for fishmeal.

**Table 3.** Global production of animal protein by source according to Faostat. N.B. Inland fish production (in italics) is for comparison and does not contribute to the table total.

protein source	global production 2007 (tonnes)	%
fish (all sources)	156 371 774	36.58
pig meat	99 211 931	23.38
chicken meat	75 826 354	17.87
cattle meat	59 851 860	14.10
*inland fish*	*10 034 537*	*2.36*
sheep meat	8 303 867	1.96
turkey meat	5 868 167	1.38
goat meat	4 828 237	1.14
14 others (each contributing less that 1%)	15 258 112	3.59
total	424 415 484	100

Fish from inland waters can be extremely important to local food security as compared with other sources of animal protein. For example, the Lower Mekong basin has a population of more than 60 million people. Inland capture fisheries yield in the region is about 2 million tonnes per year and 1 million tonnes of fish is equivalent to 1 200 000 big buffaloes or 16–17 million pigs. In Laos inland fish contributes 29 kg per person per year (48% of animal protein) and in Cambodia 37 kg per person per year (79% of animal protein; Hortle 2007).

## 4. Key features of inland waters

Fish inhabit most inland water ecosystems. The ecology of the many species, and to a large extent the methods by which they are exploited, are determined by the ecosystem and habitat characteristics. The drivers operate in different ways and diverse approaches need to be taken to their management. The main types of inland waters are as follows.

### (a) Rivers

Rivers are open, linear systems with numerous small headwater streams that depend mainly on external nutrient inputs. In headwaters, food webs are based on organic matter that is progressively degraded by invertebrate and micro-organism activity along the course of the channel (river continuum concept; Vannote *et al*. 1980). Significant drivers are the degree of deforestation, and agriculture practice in the vicinity of the river. In lowland rivers, nutrient dynamics involve material deposited on the floodplain. There is a seasonal shift in ecology associated with seasonal flooding (the flood pulse concept; Junk *et al*. 1989). Floodplains are of particular importance to the breeding, feeding and growth of many species of fish and catches from any particular system are closely correlated to the degree to which the floodplains were flooded in preceding seasons.

### (b) Lakes

Lakes are closed systems consisting of a defined body of water. Lake ecology is stable relative to rivers. Some lakes may become severely reduced in area or even dry out when flows are reduced, as, for example, Lake Chad or the Aral Sea.

Lakes are classified according to their nutrient richness—oligotrophic lakes being the lowest in nutrients and the least productive, and eutrophic lakes being high in nutrients and highly productive. Changes in water quality are the major driver of lake ecology and shifts in water transparency, dissolved oxygen regimes and resident organisms occur with nutrient enrichment (eutrophication). Oligotrophication, reversion to lower nutrient status, may occur if nutrient inputs are reduced. Pollution from other sources, and sedimentation, are additional pressures.

### (c) Reservoirs

Reservoirs, especially those with short retention times, are sensitive to changes in flow regime in inflowing rivers and may become severely reduced in area at times when the dam is opened for electricity generation or water abstraction. Rapid fluctuations in water level (daily due to hydropeaking) are a particular problem in reservoirs, so one of the main drivers of reservoir ecology is the nature of the dam operation.

### (d) Wetlands

Wetlands are primarily extensive shallow swampy areas often associated with river or lake systems as riparian floodlands. They often vary in area seasonally and depend on local rainfall, discharge from inflowing rivers, groundwater or on rising lake levels. They are usually very productive and support populations of fish that are highly adapted to the generally difficult environmental conditions of wetland habitats. Wetlands are one of the most threatened of environments.

Rice fields constitute man-made, temporary wetlands and account for over half of total wetland area in Asia. They are colonized by fish during the wet season and support high levels of fisheries production (Nguyen Khoa *et al*. 2005; Hortle *et al*. 2008).

### (e) Coastal transitional waters

Transitional waters include river estuaries, coastal deltas, coastal lagoons and inland mangrove systems. They are often integrated into complexes of floodable coastal wetland, and permanent lagoons and channels.

The ecology of the fishes depends on salinity and the main direct drivers of fisheries production are changes in flow regimes (freshwater input) leading to ingress of saline marine waters, pollution, and land reclamation and associated loss of wetlands, all leading to reduced fishery production.

### (f) Magnitude of area of inland water resources

The relative magnitude of the main categories of fresh waters (excluding transitional waters) in the various continents is shown in table 4. Globally, there are 304 million natural lakes that cover 4.2 million km^2^ (Downing & Duarte 2009). The land area covered by constructed lakes and impoundments is 335 000 km^2^ (77 million impoundments); 76 830 km^2^ of this area are farm ponds. The figures for wetland area are considered underestimates.

**Table 4.** Distribution by continent of surface freshwater resources. Note the figures for lakes, reservoirs and rivers are given in cubic kilometres whereas measures of yield are generally given relative to hectares.

	Africa	Europe	Asia	Oceania	N. America	S. America
large lakes (km^3^)	30 000	2027	27 782	154	25 623	913
rivers (km^3^)	195	80	565	25	250	1000
reservoirs (km^3^)	1240	422	1350	38	950	286
wetlands (marshes, swamps, mires, lagoons, floodplains, km^2^)	341 000	‘Eurasia' 2 075 000 (925 000 natural wetlands, 1150 rice fields)		4000	180 000	1 232 000

## 5. Key features of inland fish and fisheries

### (a) Characteristics of fish assemblages in inland waters

#### (i) Numbers of fish species

Fish assemblages in inland and coastal waters tend to be highly complex. In rivers the number of species is strongly correlated with basin area (Oberdorff *et al*. 1995). The number of species in individual river systems ranges from tens in small basins to over 1000 in large systems such as the Amazon or Mekong. In lakes, the number of species is also broadly correlated with basin area (Amarasinghe & Welcomme 2002).

#### (ii) Responses of fish assemblages to fishing pressure

Multi-species, multi-gear fish assemblages and fisheries in inland waters respond to drivers such as heavy fishing or use of illegal methods according to a model known as the fishing-down process (Welcomme 1999). This predicts that, with increases in fishing pressure (effort), the larger individuals and species will be successively reduced and even lost from the fishery (overfishing of species) until only the smaller species remain to form the basis for the fishery. Because smaller species are generally more biologically productive, and many of the larger species are fish-eating predators, production of the fish assemblage as a whole responds, so the level of catch remains the same over a considerable range of fishing pressure. Excessive fishing may reduce the functioning of the fish community (community overfishing).

### (b) Fishing gear

Fishers respond to the diversity of habitats and the large number of species, life stages and behaviour, and seasonality of the systems by developing a range of gears adapted to the capture of all species and life stages throughout the year. Up to 150 different gears are described by Deap *et al*. (2003) for a large river such as the Mekong.

## 6. Fishing and people

### (a) Inland fisheries as part of a diversified livelihood strategy

Inland fisheries differ fundamentally from their coastal counterparts in the very diversified and complex forms that inland fishing can take within the livelihood of the fisher households. Indeed, for many local populations, inland fishing is only one economic element within the diversified matrix of activities that constitute their livelihoods strategy.

### (b) Socio-economic importance of inland fisheries

The socio-economic importance of inland fisheries and their role in rural economies in developing countries are often underestimated. Inland fisheries have been perceived as ‘*backward, informal and marginal*’ economic activities (Platteau 1989) and are poorly integrated into national or local decision-making processes (Dugan 2005; Sneddon & Fox 2007; Sugunan *et al*. 2007).

Recent studies show that the true situation may be very different. It was estimated that more than 56 million people were directly involved in inland fisheries in the developing world in 2009 (BNP 2009). This number is larger than the estimated 50 million people who depend on the same activities in coastal areas.

The great majority of inland fisherfolk are engaged in the ‘small-scale’ sector, which ranges from family-based artisanal units operating with or without dugout canoes in small ponds or lakes, or along tributaries or larger river channels, to commercial enterprises with motorized and well-equipped boats fishing in larger lakes and reservoirs. Furthermore, the vast majority of the households that depend on inland fisheries are farmers or fisher–farmers who have traditionally engaged in seasonal farming and fishing.

The inland fisheries post-harvest sector generates particularly important economic opportunities for women and it is estimated that 54 per cent of the people involved in small-scale fisheries are women.

### (c) Role of small-scale inland fisheries

#### (i) Food and nutritional security

Fish play a particularly important role in improving the nutrition of millions of people in the world (table 3). Not only are they a source of protein but they also provide vitamins, minerals, fatty acids and other micronutrients essential to a healthy diet (Roos *et al*. 2007*a*,*b*). Small-scale fisheries play a critical role in the food security of producers and their families, but also provide for other consumers. Inland fish is traded far afield from local ‘inland’ markets, and a substantial part of the catch may be consumed by coastal urban dwellers.

#### (ii) Cash income generation

One of the most important contributions to the livelihood of millions of people is the role of inland fisheries as a source of cash for households, not only for families of full-time fishers but for an unexpectedly large number of rural households that live close to water bodies and engage in fishing activities for only a few weeks or few months each year (e.g. table 5).

**Table 5.** Contribution of fishery to households' cash income (US$/household/year) in different parts of the Zambezi basin, compared with other activities (% of total household income). From Turpie *et al*. (1999).

	Barotse floodplain	Caprivi-Chobe wetlands	Lower Shire wetlands	Zambezi delta
cattle	120	422	31	0
crops	91	219	298	121
fish	180 (43%)	324 (28%)	56 (13%)	100 (39%)
wild animals	6	49	1	0.4
wild plants	24	121	48	29
wild foods	0	11	7	4
clay	2	0	8	0.1

Fishing in floodplains or along rivers or lake can be operated all-year-round and offers households the possibility to generate revenues on an almost daily basis. Fishing plays a critical role as a ‘bank in the water’ (Béné *et al*. 2009) for local populations that largely rely on this activity to access cash quickly.

#### (iii) Labour buffer function

The most critical contribution of inland fisheries is its role in the provision of labour for unskilled workers who often appear to rely heavily on fishing and related activities such as fish processing for their livelihood. The common pool nature of small-scale fisheries allows poor people to engage more heavily in this activity to sustain their lives.

#### (iv) Safety net function and coping strategy in subsistence system

Small-scale fisheries also play a role as a ‘safety-net’ in that fishing can provide alternative or additional sources of income, employment and food for the poor and near-poor households whose livelihoods have been temporarily reduced or affected by unexpected shocks or in periods of individual or collective economic crisis.

#### (v) Rent and FOREX generation

The capacity of inland fisheries to generate rent and foreign exchange earnings (Valdimarsson 2003) is limited to very few fisheries, the best example being the Lake Victoria Nile perch fishery that generates more than US$250 million annually for the three riparian countries (Cowx 2005).

## 7. Drivers external to the fishery

Inland waters have suffered the most intense human-induced impacts of all ecosystems over the past 100 years. As a consequence, freshwater fishes have become threatened by a wide array of factors that seem to be the underlying cause of the decline of many fisheries. These issues can be broken down into fishery-related and environment- or watershed-related problems.

### (a) Fishery-related issues

Exploitation is one of the key drivers affecting inland fisheries. In developed countries, inland fisheries are exploited mainly by recreational fisheries. In developing countries, exploitation is largely for food (Welcomme 2001), although recreational fishing is developing as part of the tourism sector (Cooke & Cowx 2004).

The general effects of heavy fishing pressure are to reduce the abundance of desired species (reducing the value of the catch) and affect the fish population or community structures (size and species). While overall production from the fishery is generally not compromised, the quality and value of the fisheries shift towards lower-value products that are consumed locally. An important aspect of many inland fisheries is therefore not sustainability of the total catches but determining what kind of fishery management aims to achieve. The trade-offs between sustaining catches of larger higher-value species versus supplying cheaper fish to the generally more numerous underprivileged (Cowx 1998*a*) are discussed in §8*a*.

Direct conflicts often exist between commercial and recreational fishing because they exploit the same resource base, although many studies indicate that commercial and recreational fisheries can coexist (see Hickley & Tompkins 1998). When commercial and recreational fisheries compete, the allocation of the harvest is generally in favour of recreational fishing in industrialized nations; the opposite is true for developing countries.

### (b) Environmental and watershed-related issues

The greatest threats to inland fisheries come from outside the fisheries sector. Aquatic resources are subject to numerous anthropogenic perturbations (Cowx 1994; Cowx & Welcomme 1998), which have caused shifts in the status of the fisheries and a general decline in the yield. Fisheries are not generally considered of sufficiently high priority or value relative to the competing uses, and thus suffer in the face of economically and socially higher priorities, such as agriculture, hydroelectric power production or water sports.

The major drivers external to the fishery are listed in table 1 and include:

— modification of environmental form and function simplifying the environment and eliminating critical habitats;
— dams and barrages blocking passage to fish and modifying flows;
— land recovery, drainage, flood protection reducing flooded area and eliminating critical wetland habitats;
— industrial, agricultural and urban water abstraction altering the amount and timing of flows;
— land-use practices, including forestry, changing run-off and sedimentation;
— degradation of water quality through pollution and eutrophication; and
— recreational use and navigation.

## 8. Governance, institutions, management systems and strategies

### (a) Governance and access regimes

There is a wide range of access regimes and fishing right systems in inland fisheries. In most cases they remain public resources but responsibilities for management are increasingly being devolved to private individuals or groups/local communities.

The claim that small-scale fisheries in the developing world are ‘open access’ resources (e.g. Panayotou 1982; Bailey & Jentoft 1990; Machena & Kwaramba 1997) does not reflect reality. Very few inland fisheries are de facto open access. Most are linked to some form of management system at the local/community level (Fay 1989; Thomas 1996; Béné *et al*. 2003).

### (b) Current difficulties for the management of inland fisheries

The diversity of inland fisheries is to be found in their ecology as well as the social and institutional settings under which they operate. There is considerable uncertainty in the processes that govern their dynamics. Because small-scale fisheries are affected mainly by external processes, unpredictable institutional and policy environments are sources of constant uncertainty and threat. Water allocation policy and investments, water flows, pollution and climatic variability are dominant drivers of many inland fishery systems. Faced with such challenges, conventional fisheries management has generally been irrelevant as a basis for sustainable development.

### (c) Management strategies

Inland fisheries tend to evolve along a cline from initial emphasis on food production, through recreation, to aesthetic and nature conservation (Arlinghaus *et al*. 2002; Cowx *et al*. 2010). The position of any fishery along this trajectory varies most markedly between developed and developing countries (table 6). Fisheries management in industrialized countries focuses almost exclusively on recreation and conservation, whereas developing countries still focus on food security, although the emphases on recreational fisheries (Cowx 2002) and conservation (Collares-Pereira *et al*. 2002) are increasing as a result of globalization (Cowx *et al*. 2010).

**Table 6.** Different strategies for management of inland waters for fisheries in developed and developing countries. From Welcomme (2000).

	developed (temperate)	developing (tropical)
objectives	conservation/preservation	provision of food
	recreation	income
mechanisms	recreational fisheries	(commercial) food fisheries
	habitat rehabilitation	habitat modification
	environmentally-sound stocking	enhancement, e.g. through intense stocking
	intensive aquaculture	extensive, integrated, rural aquaculture
economic	capital-intensive	labour-intensive

Fisheries management can be broken down into three major domains: management of the fish assemblages; management of the fishery; and management of the environment. Which of these domains predominates depends on the type and location of the fishery. Natural lake fisheries, for example, tend to be regulated mainly by management of the fishery; enhanced fisheries in dams and reservoirs tend to concentrate more on management of the fish; and fisheries in rivers and estuaries are predominantly managed through control of the environment.

#### (i) Management of the fish

A variety of techniques are used to improve production of fish species favoured by commercial or recreation interests, to make up for shortfalls in production arising from overfishing or environmental change, to enhance the potential yield from a particular water body or for conservation initiatives (Cowx 1994, 1998*b*; Welcomme & Bartley 1998). They include:

— stocking natural waters to improve recruitment, and bias fish assemblage structure;
— stocking to maintain productive species;
— introduction of new species to exploit underused parts of the food chain or habitats;
— elimination of unwanted species; and
— construction of biased and selected faunas.

#### (ii) Management of the fishery

In addition to direct intervention on the fish populations/communities, fisheries are usually controlled by enforcement of various regulatory constraints to prevent the overexploitation of the resources and maintain a suitable stock structure (table 7). Irrespective of the regulation measures, the fundamental problem usually lies with intense fishing pressure brought about by open access to the fishery resources. Restricting access is, however, not a simple solution because many fisheries are multi-gear, multi-species and complicated by social issues, such as traditional use rights and family obligations.

**Table 7.** Comparison of tools to regulate fishing practices in commercial and recreational inland fisheries. From Cooke & Cowx (2006).

regulatory tool	commercial fisheries	recreational fisheries
closed areas	protected areas and nursery habitats	protected areas and nursery habitats
closed season	linked to spawning periods or vulnerable periods during migration	usually linked to spawning periods
catch limit	occasionally quotas	bag limit
effort regulation	licensing	partially in some jurisdictions (e.g. UK)
type of gear	to minimize damage to stocks through, for example, mesh size or highly efficient, destructive gears	usually only in specialist fisheries
size of fish	minimum size limits usually linked to size at maturity	minimum size retained in some fisheries
species of fish	occasionally quotas	at specific times and in specific places

In many fisheries in the world, management is wholly under the control of a centralized authority that regulates effort, through access or catch regulations. This can lead to social inequity by denying access to some. Centralized authorities have also proved largely ineffective because they cannot respond to the fluctuating nature of inland fishery resources and enforce regulations in highly dispersed, multi-species, multi-gear fisheries across huge areas. There is a growing tendency worldwide to charge fishing communities with the management and improvement of their resource (Welcomme 2000; §7*a*).

#### (iii) Management of the environment

Major challenges for inland fisheries managers and stakeholders relative to the environment are: (i) to defend the interests of the fisheries stakeholders by interacting and making alliances with other interested parties; (ii) to seek to limit damage to aquatic ecosystems; and (iii) to promote rehabilitation activities. A number of key strategies are promoted, usually to address one or several problems, which may be grouped under five main actions:

— reserves/refuge areas;
— pollution control and prevention;
— environmental flows;
— freedom of passage; and
— rehabilitation of degraded habitats.

#### (iv) Special needs of international river/lake basins

Many rivers and lake basins lie within the territories of more than one country. Fish often migrate from one country to another for breeding, feeding or refuge. Human activities in one country can also affect those of others. More seriously, impacts of pollution, water abstraction and damming for power generation and irrigation are transmitted downstream in river basins, potentially damaging fish stocks, or in the latter case blocking migratory routes for fish.

Common approaches need to be adopted for their management using the ecosystems (river or lake basin) approach. Many international mechanisms for such collaboration exist in the form of river and lake basin commissions, but these usually address developmental issues such as water supply, power generation or navigation, and rarely consider fisheries.

#### (v) Management models

A number of models have been developed to assist in the assessment and management of inland fish resources. Many of these were derived from models designed for marine fisheries on unit stocks. Some of these are adequate for the management of single-species fisheries in large lakes such as the Nile perch fishery of Lake Victoria, but on the whole do not perform satisfactorily in the more diffuse multi-species multi-gear fisheries of rivers and floodplains. As a consequence, a series of models have been derived to describe the performance of exploited fish assemblages. These are needed not only for the assessment and management of the fishery itself but must provide information on the impacts of any environmental changes on the fishery, especially riparian wetland drainage and damming. In view of the continuing demands on water for uses other than fisheries, models that guide the setting of discharges for environmental flows (see §7*c*) are especially urgent. The problem is that, although such models are appropriate and useful, it is difficult to act on the management advice they generate because of poor management and enforcement capacity.

### (d) Valuation of fisheries

#### (i) Attempts at valuation

It is widely acknowledged that in most parts of Africa and Latin America, and to a lesser extent in Asia, it is extremely difficult to make any accurate and up-to-date assessment of the economic value of small-scale fisheries activities. A large number of recent works underline the high potentials of small-scale fishing activities for economic development (e.g. Cowx *et al*. 2004; Neiland & Béné 2006; Sugunan *et al*. 2007).

Neiland & Béné (2006) attempted to address the lack of valuation for inland fisheries and some studies confirm the substantial values of inland fisheries. For instance, various attempts to value the Mekong fisheries have been reviewed by Hortle (2009), and Baran *et al*. (2007) estimated that the commercial value of the Lower Mekong fisheries is worth between US$550 120 and US$1 796 560 per year at first landing.

One of the major limitations of the various studies is that they often only account for the monetary value of the catch on local markets. In fact the actual value of these small-scale fisheries goes far beyond this market value, highlighting in particular the critical role that the sector plays in terms of food security, sources of cash and employment for resource-poor local communities in remote rural areas (e.g. Béné *et al*. 2009).

#### (ii) Recreational fisheries

Recreational fisheries are the dominant use of fish resources in inland waters in the North and South temperate zones, particularly Europe, North America and Australia. The sector is also experiencing explosive development in many transitional economies in Asia and Latin America and a few countries in Southern Africa (Angola, South Africa, Zambia). The economic potential of recreational fisheries is very high. Direct income is generated from the sale of fishing licences, which may have to be paid to the owner of the fishing rights whether this is a public or private entity. The sector also has a considerable secondary income generating effect through producers and sellers of fishing equipment, bait providers, boat renters, guides, lodge owners, travel agencies, restaurants, boat constructors, producers of books, magazines, documentaries and digital information on sports fishing, and producers of stocking material.

#### (iii) Ecosystem services associated with inland fisheries

A number of ecosystem services are associated with inland water fisheries as defined by Holmlund & Hammer (1999) and the United Nations 2004 Millennium Ecosystem Assessment. Fisheries management strategies should aim to conserve the full range of services if possible, although in many circumstances some will be awarded higher priority than others.

#### (iv) Energy use

Inland fisheries are characterized by a relatively low dependence on fossil fuels so the carbon footprint of the sector is remarkably low compared with other food production systems. Fisheries use energy in three main ways: the manufacture of gear; movement to and from the fishing site; and preservation and post-harvest transport.

*Manufacture of gear*. Many of the gears used are made of locally derived materials, although the growing and widespread use of gill-nets and other gears made from artificial fibres does have some carbon cost.

*Movement to and from the fishing site*. Many fishers operate from the bank or in shallow waters so they do not need fishing craft. Where craft are used they are usually small hand-propelled canoes or sometimes use sail.

*Post-harvest preservation and transport*. Fish products are conserved by a variety of means. Where electrical power is available, lake and river fishers use ice to conserve the catch on their journeys to market. Where power is not available, most of the artisanal post-harvest sector still uses traditional conservation techniques such as sun-drying, salting and smoking for round-fish conservation, and fermented pastes and sauces for smaller fish.

## 9. Aquaculture and culture-based fisheries

### (a) Inland fisheries aquaculture interactions

**Figure 3.**
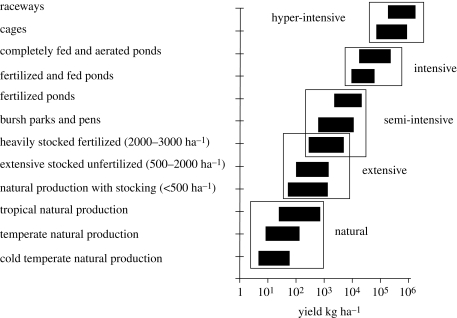
Production for different capture and culture systems. Adapted from Welcomme & Bartley (1998).

Capture fisheries harvest wild aquatic animals held in some form of common ownership, while aquaculture involves the active rearing of aquatic animals held in private ownership. There is a continuum of inland fishery systems using varying degrees of enhancement and management that fall between true wild capture fisheries and true aquaculture (figure 3).

### (b) Culture-based and enhanced fisheries

Releasing fish spawned and bred in aquaculture systems into natural populations can add to total production and population abundance (Lorenzen 2008). However, such measures may impact negatively on the wild population through density-dependent responses and introgression of hatchery stocks often characterized by reduced genetic diversity and fitness (Lorenzen 2005). There are also issues relating to the possibility of disease transmission, although in many cases aquaculture stocks may be healthier than wild stocks.

Strategies for stocking also vary according to the water body and the manner of stocking or enhancement. In some cases fish are stocked for almost complete recapture, such as in seasonal irrigation reservoirs and water bodies that are considered culture-based fisheries. Elsewhere, in permanent water bodies and large reservoirs, stocking would have a minimal impact on overall fishery recruitment, and a strategy of stocking species that will breed in the water body and contribute to recruitment is favoured.

The enhancement of fisheries usually involves some form of ownership over what were previously open access fisheries. As a result there are often social problems with enhanced/culture-based fisheries in developing countries, due to aspects of rights to fish and access. Furthermore, water management of the water body may not prioritize fisheries and thus the fishery/culture-based fishery production may not be optimal, or even be severely impacted by such externally imposed factors as the draining down of irrigation water bodies. Aquaculture concessions granted to a user or user group may resolve access issues, but in some cases, the concession may marginalize traditional users and the benefit may be limited to a few individuals.

### (c) Capture-based and self-recruiting aquaculture

Capture-based and self-recruiting aquaculture are culture systems based on the use of broodstock, fingerling or fry captured from the wild or recruiting naturally into the culture facility (i.e. there is no system of captive breeding). All aquaculture was originally based on wild stocks and was only liberated by the development of artificial breeding techniques in the 1950s.

Capture-based and self-recruiting aquaculture remain strongly dependent on the productivity of wild fish stocks and are only viable in the longer term where fishing pressure on the fry remains within the limits imposed by the ability of wild populations to compensate for removal of early life stages through density-dependent processes.

### (d) Can aquaculture substitute for declining capture fisheries?

Aquaculture development is often promoted to mitigate for real or perceived declines in inland fisheries and their contribution to rural livelihoods. However, fisheries and aquaculture are very different activities, and it is not usually possible to simply replace fisheries with aquaculture. The main reasons for this are:

— Fisheries exploit common pool resources. Their exploitation requires access to the water body and resource, but does not require land and can operate with very little capital. Aquaculture is the farming of aquatic organisms owned privately, and it requires private rights to land or an area of water, and substantial capital, especially if production is intensified.
— Aquaculture is a farming activity, and requires inputs such as seed fish, feed and/or fertilizer, if yields are to be raised over and above ‘natural’ levels.
— Fisheries present livelihood options for a wide range of people, but particularly the poor. Aquaculture is often taken up predominantly by better-off households, although successful uptake by the poor is possible.
— To develop, aquaculture needs supply industries, in particular, seed production and distribution networks. Where such networks do not exist, substantial support may be required for their development.
— Aquaculture produces a small number of mostly large-bodied species that provide high quality protein, but may not replace the diversity of micronutrients (especially calcium and vitamin A) available from small, wild fish eaten whole.
— There are cultural barriers to the development of aquaculture and its uptake by particular groups. For example, because aquaculture is essentially a farming activity, it is rarely taken up by full-time fishers.

For these reasons, it must not be assumed that aquaculture development is necessarily a suitable compensation measure for loss of fisheries yield.

## 10. Potential effects of climate change (adapted from Halls 2009)

Climate change is likely to affect inland fisheries through several mechanisms.

### (a) Temperature

Higher temperatures reduce oxygen solubility in water but can raise the oxygen and food intake demand of fish as their metabolic rates are raised. Associated rises in gill ventilation rates can lead to increased uptake of aquatic pollutants, potentially rendering the flesh unfit for human consumption. Higher water temperatures can also favour the survival of parasites and bacteria. All these responses combine to potentially reduce fish survival, growth and reproductive success both in wild populations and aquaculture systems (Ficke *et al*. 2007). Similarly, many species in temperate regions have characteristic temperature ranges in which they live and breed and rises in temperature may result in species being displaced to higher latitudes to be replaced by species preferring higher temperatures.

### (b) Hydrological impacts

In rivers, increasing flows during the flood season will translate to more extensive and prolonged floodplain inundation, potentially increasing overall system productivity including the fish component (Welcomme 1985; Junk *et al*. 1989). Longer, more extensive floods are likely to provide greater and more prolonged feeding opportunities for fish. Improved growth can favour survival and reproductive potential (fecundity). Changes to the timing of flows also have the potential to disrupt spawning behaviour (Welcomme & Halls 2001).

The dry season is a period of great stress to many river fish species arising from diminished feeding opportunities and water quality, and elevated risk of predation or capture. Fish survival during this period is therefore likely to be density-dependent. Increased precipitation and water availability during this period might favour fish survival and ultimately exploitable biomass, while drier conditions would have the converse effect (Halls & Welcomme 2004).

The combination of reductions in river flow and sea level rise may change salinity profiles in river deltas and lead to greater upstream salinity intrusion. These changes may displace stenohaline (narrow salinity tolerance) species further upstream and increase the upstream range and biomass of euryhaline (wide salinity tolerance) species, including those that depend upon brackish water environments to complete their life cycles.

Perhaps the greatest impact will be in the conversion of snow- and glacier-fed rivers to rain-fed rivers as the permanent ice in many mountain regions is eroded. This will change the hydrological characteristics of such rivers fundamentally, altering their seasonality and the evenness of the food regimes.

### (c) Watershed/basin level impacts

Careful consideration will have to be given to both planned and autonomous adaptive coping strategies pursued by the agricultural sector. Less predictable flooding patterns and reductions in dry season flows may force small-scale farmers to build makeshift levees to protect their crops from flood damage and to rely increasingly on surface water bodies to meet their irrigation needs. Planned adaptation may favour the construction of large-scale storage reservoirs, flood control embankments and irrigation schemes with an associated increase in withdrawal of water from the aquatic ecosystems, which impact negatively on the fisheries sector by obstructing fish migrations and diminishing dry season habitat availability and quality (Halls *et al*. 1998, 1999).

## 11. Future of inland fisheries

Fisheries in Asia are very heavily exploited and have very little apparent room for expansion by better management. In Africa fishing pressure, although increasing, is still below the level experienced in Asia so there still may be some potential for expansion. The economic value of small-scale fisheries in Africa could be doubled or tripled simply by improving post-harvest processing techniques. In Latin America, fisheries appear relatively less heavily exploited than in Asia, with few signs of fishing down at the community level, although some individual stocks are under pressure. Inland fish resources in Europe, North America and Australia are exploited more for recreational than consumptive purposes, and often managed to meet conservation objectives (Cowx *et al*. 2010). As a result production for food is declining.

The significance of current reported catches is difficult to assess. It is assumed that actual catches have been at a maximum level for some time, although real increases are still occurring in some fisheries. Increases in reported catch are mainly because of improved reporting of hitherto unrecorded sources of inland fish, such as small-scale artisanal and subsistence yields, or yields from rice fields. It is impossible to predict at what level reported and actual catches will merge, if ever, although it is clear that present actual production exceeds the 10 million tonnes estimate by a large margin.

Better understanding of the significance of inland fisheries resource may influence the direction of general development policies for aquatic systems, in particular in relation to further hydropower and irrigation investments. The greatest risk, particularly in rivers, coastal lagoons and estuaries and river-driven lakes, is modification of flow regimes by water abstractions and power generation, principally through damming. Climate change is likely to exacerbate the situation arising from adaptive strategies such as flood control, and increasing demand for water for irrigated agriculture. The risks of losing catch are also increased by other forms of environmental damage such as draining of seasonal riparian wetlands and river channelization. The assumption that better identification of the role of inland fisheries in livelihoods and food security would result in the sector's needs being considered when planning new civil works on rivers has so far been unjustified. As a result, losses of inland fishery production can be anticipated in many rivers, lakes and wetlands.

One method to mitigate for this loss is to develop improved fishery enhancements in the inland waters that remain after the present wave of modifications. Fishery enhancement was popular in the 1980–1990s and achieved notable successes in increasing inland fish production in many countries. Unfortunately, current trends seem to indicate that use of public funds to support large-scale stocking is not acceptable in the existing financial climate, so the practice has declined in several countries (de Silva & Funge Smith 2005). Nevertheless, knowledge of the technique is still available and may well re-emerge as an option if food security becomes an issue.

In summary, inland fisheries are an important source of cash and protein food, particularly in poorer countries where its products are readily available to the population. Yields at present are probably well in excess of 10 million tonnes per year, but the prognosis for the future is far from good with many of the external drivers reducing the amount being caught from many wild fisheries. This will almost certainly result in issues of changing supply and availability to some rural areas which remain dependent upon inland fisheries as a food source.
